# What moves large grazers? Habitat preferences and complementing niches of large herbivores in a Danish trophic rewilding area

**DOI:** 10.1007/s00267-025-02164-8

**Published:** 2025-04-25

**Authors:** Bent Johann Rech, Robert Buitenwerf, Roberto Ruggiero, Jonas Trepel, Matthias Waltert, Jens-Christian Svenning

**Affiliations:** 1 Institute for Social Ecology, BOKU University, Schottenfeldgasse 29, 1070 Wien, Austria; 2 Department of Conservation Biology, University of Göttingen, Bürgerstrasse 50, 37073 Göttingen, Germany; 3 Center for Ecological Dynamics in a Novel Biosphere (ECONOVO), Department of Biology, Aarhus University, Ny Munkegade 114, 8000 Aarhus, Denmark; 4 Department of Bioscience and Territory, University of Molise, 86090 Pesche, Isernia Italy

**Keywords:** Habitat selection, Large herbivores, Megafauna, Remote Sensing, Restoration, Rewilding

## Abstract

Large herbivores (≥45 kg) fulfill key ecological functions. Since the Late Pleistocene megafauna diversity and abundances have declined sharply, with profound consequences for ecosystems. On this background the concept of trophic rewilding has emerged and is increasingly applied to restore natural disturbance regimes and trophic interactions, ultimately aiming to recreate self-sustaining, dynamic and diverse ecosystems. Effects of such efforts (e.g., more heterogeneous habitats) are evident, but herbivore space use, and the resulting distribution of effects on vegetation remain poorly understood. Here, we examine habitat selection of semi-feral water buffalos (*Bubalus bubalis*), horses (*Equus ferus*) and cattle (*Bos taurus*) in a Danish rewilding area. We modelled space use with remote sensed covariates, reflecting resources (vegetation greenness, distance to water) and infrastructure (distances to fences, paths, shelter). Seasonal differences and former land use were tested separately. We found large-herbivore space use to shift seasonally, reflecting food and water availability, and to be influenced by infrastructure and former land use. Horses reacted less to vegetation greenness and water than the two bovids. Cattle selected for green vegetation in summer, while buffalos showed the strongest association with water bodies. Overall, the three semi-feral herbivore species diverged in their habitat use both spatially and seasonally. This can be expected to translate to variable and complementary ecological impacts such as grazing, physical disturbances and habitat engineering. Such variable space use likely increases habitat heterogeneity and species richness. We therefore suggest that a diverse large-herbivore guild is key, both to understanding megafauna ecology and for successful rewilding efforts.

## Implications for practice

- Large herbivores complement each other in their habitat use, therefore reintroducing more diverse herbivore assemblages likely maximizes desired ecological impacts
- Herbivore space use varies seasonally with larger parts of the area getting utilized in winter, underlining the importance of year-round compared to summer grazing
- Anthropogenic factors such as artificial water sources or infrastructure shape the space use of large herbivores and should therefore be carefully considered when designing rewilding projects

## Introduction

Biodiversity is declining rapidly and worldwide, often due to anthropogenic causes such as habitat loss, climate change, altered nutrient cycles, pollution, over exploitation and habitat change (e.g., Mazor et al. 2018; Chapin III et al. 2000). This looming 6^th^ mass extinction (Cowie et al. 2022) threatens ecosystem functioning, resilience, and potentially also the ecosystem services necessary for human persistence and sustainable development (Cardinale et al. 2012; Blicharska et al. 2019). Additionally, an ethical obligation to protect ecosystems and species, because they possess a right to existence and are immeasurably valuable regardless of their functions for humans, can be argued for (Taylor et al. 2020). However, efforts, funds and research are still lacking, and agreed upon targets have not been met (Butchart et al. 2010; Xu et al. 2021).

Humans are increasingly considered the main driver of Late Pleistocene megafauna extinctions and population declines of extant species (Svenning et al. 2024; Sandom et al. 2014; Bergman et al. 2023). Megafauna disappearance had fundamental impacts on ecosystems when formerly widespread grazing, browsing, trampling, physical disturbance and defecation ceased. Consequentally, habitat structure homogenized, competition for light increased, dispersal connectivity was crippled, nutrient cycling and distribution were profoundly altered, and engineered ecological niches and species adapted to them disappeared (Søndergaard et al. 2025; Svenning et al. 2024; Enquist et al. 2020). Extensive pastoralism, which partly mimics the functions of wild large herbivores, has been drastically declining and the size specific loss of megafauna and their ecological functions continues during the Holocene (Bocherens 2018; Davoli et al. 2024). As a consequence research and restoration increasingly engage with trophic rewilding, which can be understood as “an ecological restoration strategy that uses species introductions to restore top-down trophic interactions and associated trophic cascades to promote self-regulating biodiverse ecosystems” (Svenning et al. 2016). Such efforts showcase the ecological impacts of large and megaherbivores, including biomass consumption, nutrient cycling and redistribution, seed dispersal and increased habitat heterogeneity (e.g., Pires et al. 2018; Ripple et al. 2015; Trepel et al. 2024). Through effects such as fire regulation or increased albedo of opened habitats, large herbivores can maintain biodiversity and ecosystem functioning and support adaptations to global change (e.g., Malhi et al. 2022; Schmitz et al. 2023; Svenning et al. 2024b). While there are concerns about potentially undesired effects due to reintroducing species which are considered non-native or feral, Lundgren et al. (2024) show that not nativeness but rather functional traits shapes impact of large herbivores.

While the ecological importance of large herbivores is well documented, less is known about the space use of such animals in trophic rewilding projects. Herbivores select their habitat based on a variety of qualities like cover, forage availability, disturbance or fear (e.g., Müller et al. 2017; Burkepile et al. 2013). With impactful species like large herbivores (Trepel et al. 2024), their very habitat selection mediates distribution, intensity and frequency of ecological functions (e.g., biomass removal, defecation). So, the habitat selection of large herbivores indirectly shapes ecosystems (e.g., vegetation structure) and is thus vital to their understanding and management.

Many current projects only engage in seasonal grazing (i.e., absence of winter grazing) which affects herbivory impacts (e.g., Zielke et al. 2019). Therefore, we studied the habitat selection of three semi-feral large herbivores - cattle (*Bos primigenius taurus*), water buffalos (*Bubalus bubalis*) and horses (*Equus ferus caballus*) - in a Danish rewilding project with year-round grazing (Geding-Kasted Mose). Specifically, we hypothesize that:

1. There are seasonal differences in the selection of used areas:
  - water bodies have stronger effects in summer, when drinking and wallowing is more important.
  - used area is larger in winter, as food is sparser.

1. Large grazers select for proximity to water, to maintain hydration and temperature.
2. Large grazers select for green biomass, reflecting food quantity to forage effectively.
3. Human infrastructure (i.e., fence and paths) has no significant effects because human activity levels are low, and the large herbivores have habituated to these landscape modifications.
4. Land-use history and habitat types shape habitat structure and food availability thereby affecting habitat selection patterns of large grazers.

The first three hypotheses reflect ecologically important habitat qualities and attempt to provide new insight into their respective role in the context of trophic rewilding. The last two hypotheses specifically address if and how large-herbivore habitat selection is influenced by anthropogenic factors. Additionally, all hypotheses can yield management implications, i.e., related to the design of rewilding projects.

## Methods

### Study area

**Fig. 1:**
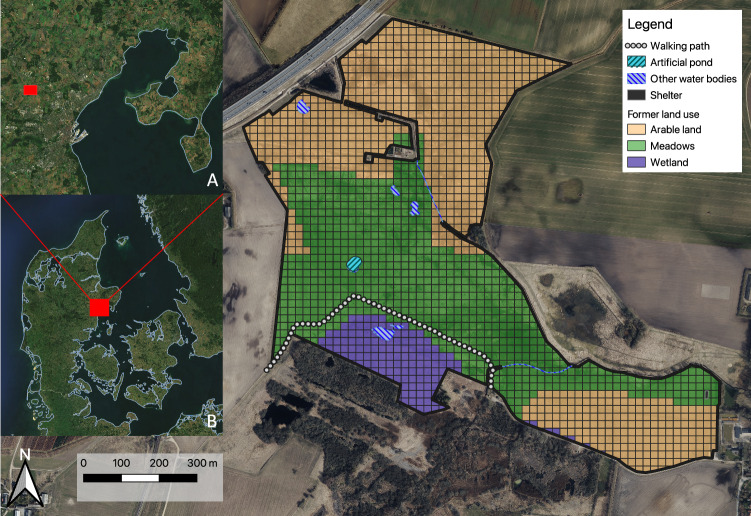
Map of the study area Geding-Kasted Mose. Showing the grid cells (20*20 m) to which all observations and covariates were attributed. Former land use (last CORINE landcover classification from 2012), paths, water bodies and the shelter are indicated (see legend). Overview map “**A**” shows the location of the study area (red box) in the North-West of Aarhus. The second overview map “**B**” shows the location of map “**A**” in Denmark, indicated by another red box

The study was conducted in the Geding-Kasted Mose trophic rewilding project in northern Denmark (56°12'43.6“N, 10°06'42.9“E). The study area comprises 17 former properties, with differing land use and vegetation histories (e.g, meadows, pastures, cropland, forest, coppices, orchards), that were acquired by the city of Aarhus and combined into the project area (Aarhus municipality). According to the last CORINE landcover classification (Büttner 2014), the study area was subdivided into (1) wetlands including bogs, marshes, and swamps, (2) former meadows and, (3) former arable fields, all in various post-use successional stages (see Fig. 1 & Supplementary Fig. S1). In wetlands Cyperaceae and Juncaceae are abundant, complemented by *Typha latifolia* L., *Phragmites australis* (Cav.) Trin. ex Steud. reedbeds, Apiaceae and expanding *Salix* spp. and *Betula* spp. Former meadows show well established, palatable grassland communities dominated by Poaceae (e.g., *Elymus repens* L., *Lolium perenne* L.) and interspersed by legumes (e.g., *Trifolium repens* L., *T. pratense* L.), which locally dominate in shortly grazed lawns. Former arable lands show bare ground and sparser vegetation cover including smaller rosette plants (e.g., *Hieracium* spp.), former crops (e.g. *Medicago sativa* L., *Triticum aestivum* L.) and thistles (*Cirsium arvense* (L.) Scop., *C. vulgare* (Savi) Ten., *Carduus crispus* L.).

Several smaller streams originate in the study area and several natural ponds and former peat digs are found (see Fig. 1). The area shows little topographic variation - elevation of ~23 m to ~32 m ASL. - and is ~135 ha in size; however, some parts (~64 ha) have been added recently (2021/22, i.e., after data collection), which is why this study focuses on the original area of ~71 ha (see Fig. 1). Further, only observations contained within the initial fence line were used in this study.

In 2016 semi-feral water buffalos, horses and cattle were released in the area, chiefly to maintain landscape openness, to facilitate the area’s function as a subterranean aquifer for freshwater extraction (Aarhus municipality, personal communication). Apart from a shelter (legally required for animal welfare regulations), a constructed waterhole (see Supplementary Fig. S1), mineral licks, yearly veterinarian examinations, occasional stocking adjustments and limited supplementary feeding, the animals live and socially organize free of human interventions (Aarhus municipality, personal communication). Due to the delineated fence, the populations can be considered closed to im- or emigration. The large herbivores in the trophic rewilding area have year-round access to the whole area. As both population size and structure are anthropogenically controlled, the “Geding-Kasted Mose” site can be classified between “minimal” and “partial” rewilding in the TRAAIL framework by Pedersen et al. (2020). Approximately ~40 cattle and ~6 horses of both sexes, and 3 castrated, male buffalos roam the study area. However, exact stocking rates and breeding practices are constantly adjusted and determined by the animals’ owner and remain partially unknown to the authors.

### Data collection and preparation

#### Dependent variable – species observations

To assess habitat selection of the three large herbivore species, animals were tracked using handheld GPS units and following a standardized protocol from 2018 to 2021: After entering the area (92% of observations between 9 am 4 pm), the first group of animals that was encountered was observed. The individuals of the group were counted from left to right until the first number on a list of randomized numbers was reached. If the group was smaller than the random number, counting continued until the number was reached. The respective animal was then observed for 60 s, and the dominant behaviour (>30 s) was selected from a list of predefined categories (Supplementary Table S1), that were simplified into feeding and non-feeding for analysis later on, as sample sizes of single behavioural categories differed strongly. The observation was assigned an approximate position in the GPS device and an observation ID. Date, time, behavioural category and group size were added. If animals moved during the observation period, the approximated mean location between the start and end position was used. After the observation of a single individual, the dominant behaviour of the group (i.e., >half of the individuals) was recorded at the approximate centre of the group, with the entry again containing observation ID, date, time, behavioural category, and group size.

This cycle of a 60 s individual observation and an instantaneous group observation was repeated for a block of 90 minutes (averaging to ~60–90 observations per block). To ensure observations of all species and to prevent oversampling the species and the group of animals were to be changed after every observation block. All gathered observations were combined in Quantum GIS (v. 3.24 Tisler; QGIS development team 2022) and duplicates or faulty records were disregarded. In total 25486 observations (of individuals and groups) were recorded and considered in this study (see Supplementary Table S2 for differences between years and species).

#### Independent variables – fence, footpath, shelter, water, wetness, greenness

To assess the role of infrastructure we used distance to the fence, the distance to the visitor footpath, and the distance to the shelter. All distances were calculated using the NNJoin plugin in QGIS (v. 3.24 Tisler; QGIS development team 2022). All distances were measured as straight lines from the centroid of the respective grid cell to the nearest respective structure, disregarding potential obstacles due to the small size and topographic homogeneity of the study areas.

All identifiable water bodies were drawn from a public orthophoto (Danish Agency for Data Supply and Infrastructure 2021). During data exploration the distance to the artificial pond exhibited differing effects compared to other water bodies. Therefore, two water variables were used: the distance to the artificial pond and the distance to all other water bodies (i.e., excluding the artificial pond).

We used the Topographic Wetness Index (TWI) calculated from a digital elevation model (DEM) based high resolution LiDAR scans for Denmark (Assmann et al. 2022). The slope (in degrees) was calculated from the DEM and converted to radiance (S_r_). After that, upslope area (U_a_) was calculated using the flow accumulation tool. Finally, the TWI was calculated using the following formula:

$${\rm{TWI}}=\mathrm{ln}\left(\left({\rm{U}}{\rm{a}}\times 20\times 20\right)/\tan \left({\rm{S}}{\rm{r}}\right)\right)$$

This equation is the natural logarithm of the upslope area (U_a_) times the grid cell dimensions (20 m × 20 m) divided by the tangent of the slope in radiance (S_r_).

As a proxy for vegetation productivity the Enhanced Vegetation Index (EVI) was calculated using Google Earth Engine and Sentinel 2 satellite imagery, which has a 5 day revisit interval and a 10 m resolution for the bands used here (Earth Engine Data Catalog 2022). EVI is described by the following formula:

$$\mathrm{EVI}={\rm{G}}\times \left(\left(\mathrm{NIR}-{\rm{R}}\right)/\left(\mathrm{NIR}+{{\rm{C}}}_{1}\times {\rm{R}}-{{\rm{C}}}_{2}\times {\rm{B}}+{\rm{L}}\right)\right)$$

Where NIR is the near infrared band, R is the red band, C_1_ and C_2_ are coefficients for atmospheric resistance, L accounts for canopy effects, B is the blue band and usually “L = 1, C_1_ = 6, C_2_ = 7.5, and G (gain factor) = 2.5” are used as coefficients (Huete et al. 2002). Compared to the Normalized Vegetation Difference Index (NDVI), the corrections used for the EVI partly account for canopy cover and atmospheric distortion and render it less prone to saturation (Huete et al. 2002; Liu and Huete 1995). We calculated mean EVI values for each combination of season (summer from 1st May to 31st October and winter from 1st November to 30th April and study year (2018–2021). Images with cloud cover >10% were discarded.

#### Land- use history

To investigate the effects of land-use history, the latest CORINE land cover classification from before the project start (European Environmental Agency EEA 2012) was utilized to subdivide the area into three former land use types: 1. arable land, 2. meadows, and 3. wetlands (see Fig. 1). These classifications were employed to test the effects of land use legacy on animals’ space use.

For modelling habitat selection, the small-scale and detailed former properties within the study area were used as predictors.

### Data preparation and organization

QGIS (v. 3.24 Tisler; QGIS development team 2022) was used for field observation, data organization and remote sensing, except for the EVI, which was initially calculated using Google Earth Engine (Gorelick et al. 2017). For comparability and consistent resolution, the area was divided in 1783 equal 20*20 m grid cells, to which all variables were attributed. The resolution is a compromise. Due to the openness and lack of topographic variation of the study area, we are confident that the chosen resolution sufficiently accounts for the inherent inaccuracy of field-based, approximated locations. Furthermore, given the small size of our study area and fine-scale patterns (e.g., surrounding surface water bodies), we aimed for a high, yet still robust, resolution.

The animal observations were split by species (buffalo, cattle, horse), behaviour (feeding, non-feeding), year (2018–2021) and season (summer, winter). The seasonal split was based on the local climate, with the 6 coldest months (November-April) as the winter period and the other 6 months (May-October) as the summer period. Apart from the EVI, all predictors were assumed to stay constant over the course of both seasons and all four years of study. We attributed both individual and group observations to the grid cells, as we consider both valuable indicators of the spatiotemporal distribution of animals. We had to forego weighing group observations based on group size, because the anthropogenic and unequal social structures (e.g., 3 male buffalos versus ~40 cattle of both sexes and several age classes) and unknown stocking management practices could not be adequately accounted for.

### Data analysis

All statistical analyses were performed in R (v. 4.2.1; R Core Team 2022). We used generalized linear mixed effects models (GLMMs) considering the number of animal observations of a certain species and behaviour in a given cell in either winter or summer as our response variable. Additionally, we used (a) observation year, (b) coordinates of the grid cell centroid (c) and land-use history (i.e. the former properties) as random effects. We accounted for spatial autocorrelation by adding grid cell locations (i.e., centroid coordinates) as random effect. We encountered a lack of observations in areas classified as wetlands in CORINE (e.g., for non-feeding buffalos in winter), which generated very large coefficient estimates (e.g., effects >15 and standard errors >2000) when using CORINE land cover as a fixed effect. This indicates that the model was too complex for the available data, rendering it uninterpretable. Furthermore, the AIC of models with CORINE as a predictor was usually higher than for models with the 17 former properties. Finally, the CORINE land cover classification has a 100 m resolution, which is relatively coarse for our study area, and limited accuracy (Büttner and Maucha 2006). Therefore, we opted to use the 17 former properties as proxies for land-use history instead of CORINE. Given the unknown specific use history of these properties, they were treated as a random effect. To ensure interpretability and comparability, we excluded CORINE in all models and instead used it separately to test the effects of former land use with Wilcoxon tests, as CORINE was still the best indicator of known prior use.

As fixed effects we used the distances (in metres) to (1) the fence line, (2) the walking path, (3) the shelter (assessed in a separate model with the same random effects due to collinearity issues), (4) the single artificial pond and (5) all other water bodies. Models were fitted using the “glmmTMB” package (Brooks et al. 2017).

For model selection, AIC was used to balance complexity and explanatory power by comparing models relative to one another (e.g., Wang and Liu 2006). A backward selection approach was applied: The full model was reduced by one variable at a time to compare differing variable combinations by AIC. However, as this study centres around proposed hypotheses and their respective falsification, covariates were not selected purely based on AIC. Rather the criteria allowed to investigate if and how the covariates that were chosen based on prior ecological or management interests affected the model’s balance between predictive power and complexity. Thus, if a covariate did not increase AIC strongly (>5) (Burnham and Anderson 2004) it could still be used as it referred to a hypothesis. Therefore, distance to fence, the grid cell centroid coordinates, EVI and TWI became part of the model, despite not decreasing AIC for all three species. Through this procedure all variables became part of the model, while no interactions were added, as they increased AIC for the models of feeding animals in the summer. To ensure comparability, this same model structure was used for all species, behaviour and season combinations.

Posterior model checking of the resulting twelve models (three species, two types of behaviour, two seasons) was performed with simulated residuals using the DHARMa package (Hartig 2022). The overall visual fit of all models was assessed to be sufficient, and significant results of the included tests can be attributed to high sensitivity due to the large sample size (see Supplementary Figs. S2–S4).

Independent of the modelling results the effects of former land use (based on CORINE information) were analysed with Wilcoxon tests comparing the mean of observations between summer and winter for all former land use categories and all 4 study years. The number of observations per season were adjusted to account for different sample sizes by descaling the season with more observations.

Additionally, using proportion tests on every combination of behaviour and species we compared the share of occupied (i.e., >1 observations) and non-occupied (i.e., 0 observations) grid cells between seasons.

## Results

### Modelling results - Cattle

**Fig. 2:**
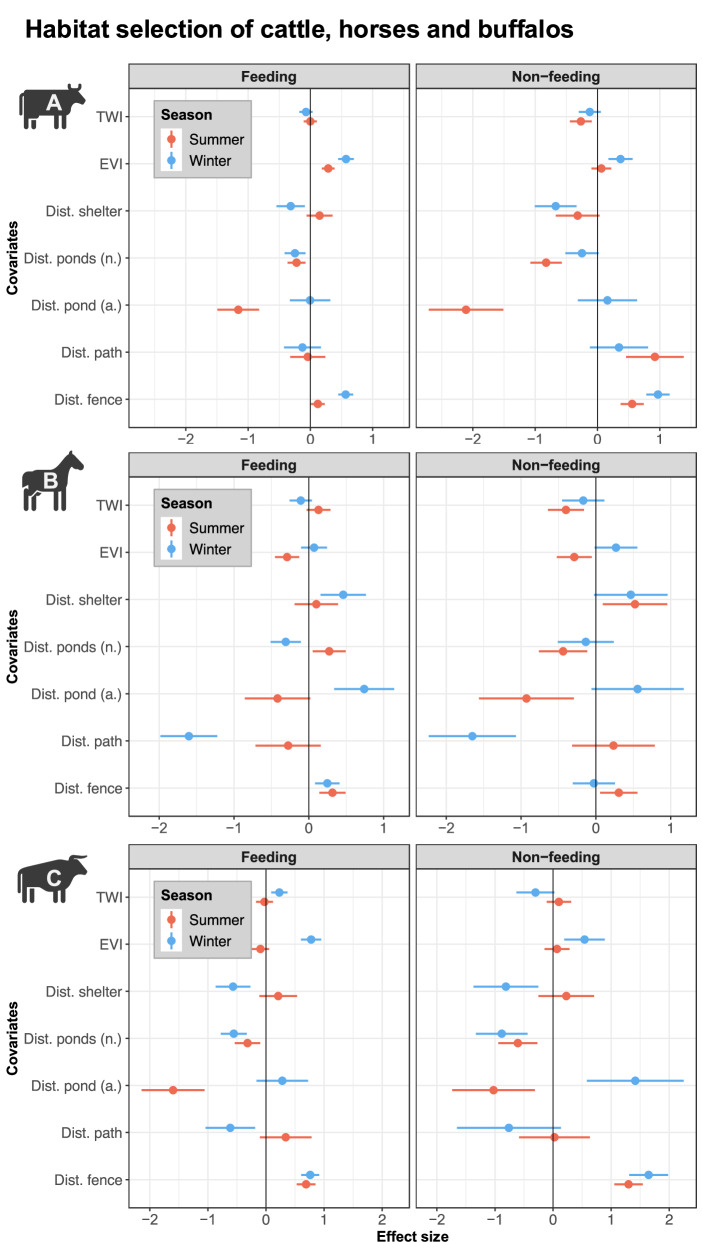
Estimated effects of covariates on the abundance of feeding and non-feeding cattle (**A**), horses (**B**), and buffalos (**C**) in summer and winter period. The central points mark the model estimate, while the horizontal line (whisker) refers to the confidence intervals (CI, 2.5–97.5). Estimates and CI that did not overlap with the vertical black line indicate a significant effect of a given covariate with values left of the line indicating a negative effect and values right of line a positive one. The figure includes the following covariates: topographic wetness index (TWI), enhanced vegetation index (EVI), distance to the artificial (a.) pond (m), to natural (n.) water bodies (m), to the walking path (m), to the fence line (m) and to the artificial shelter (m), with the later having been assessed in a separate model due to collinearity

In both seasons, feeding cattle selected for areas with comparatively more green vegetation (i.e. higher EVI), while topographic wetness (TWI) was mostly non-significant (except in summer where non-feeding cattle avoided wetter areas; Fig. 2 and Supplementary Table S3). In winter but not in summer, cattle stayed closer to the shelter, regardless of behaviour (Fig. 2 and Supplementary Table S3). In summer, cattle selected for the artificial pond, independent of whether they were feeding or not, but did not show any preference during winter (Fig. 2 and Supplementary Table S3). The effects of the distance to other water bodies were weaker, but significantly negative for all behaviour-season combinations except non-feeding cattle in winter (Fig. 2 and Supplementary Table S3). While distance to the path was mostly non-significant, the distance to the fence had significant positive effects (Fig. 2 and Supplementary Table S3).

### Modelling results - Horse

Regardless of behaviour, horses selected against EVI in summer, while effects where non-significant in winter (Fig. 2 and Supplementary Table S4). Horses did not select for proximity to the shelter in either season (Fig. 2 and Supplementary Table S4). Compared to the bovids, horses showed a weaker association and wider confidence intervals with the artificial pond and mixed effects of the natural water bodies (Fig. 2 and Supplementary Table S4). Horses avoided the fence line in both seasons but stayed closer to the path in winter (Fig. 2 and Supplementary Table S4).

### Modelling results - Water buffalos

Water buffalos selected for EVI in winter, but not in summer (Fig. 2 and Supplementary Table S5). Similar to the other species topographic wetness was mostly non-significant for buffalos. However, the only significant positive effect of TWI in all models is for feeding buffalos in winter (Supplementary Table S5). Buffalos selected for proximity to the shelter in winter, while effects were non-significant in summer (Fig. 2 and Supplementary Table S5). In summer, water buffalo selected for proximity to the artificial pond (Fig. 2 and Supplementary Table S5). In contrast to the other species, buffalos also selected for proximity to natural water bodies across the two behaviours and seasons (Fig. 2 and Supplementary Table S5). The path had weak effects on the space use of water buffalo, while the fence was avoided in both seasons and behaviour types (Fig. 2 and Supplementary Table S5).

### Seasonal shifts and land use legacy

**Fig. 3:**
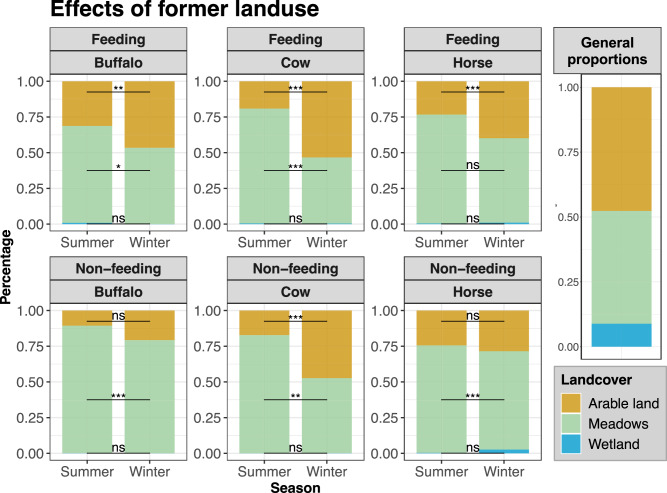
Proportional use of the three former Corine landcover classes (arable land, meadows, peatland), divided by species and behaviour and comparing summer to winter distribution from the whole study period (4 years). The lines between the bars indicate the results of Wilcoxon tests, where one or more “*” indicate a significant difference between the mean number of observations in summer and the mean number of observations in winter. The larger, single bar chart on the right shows the general proportion each landcover class has in the study area

Focusing on the CORINE classification of former land use, meadows and pastures were disproportionately favoured by all species across various behaviours and seasons. Conversely, areas previously classified as wetlands and arable land were significantly underutilized relative to their availability (Fig. 3). All species showed a seasonal shift with significantly less observations in former meadows in winter, while the means of observations in former arable land increased simultaneously (Fig. 3). However, even in winter former arable land and wetlands often held less observations than expected for their respective share of the area if space use was distributed randomly (Fig. 3).

**Fig. 4:**
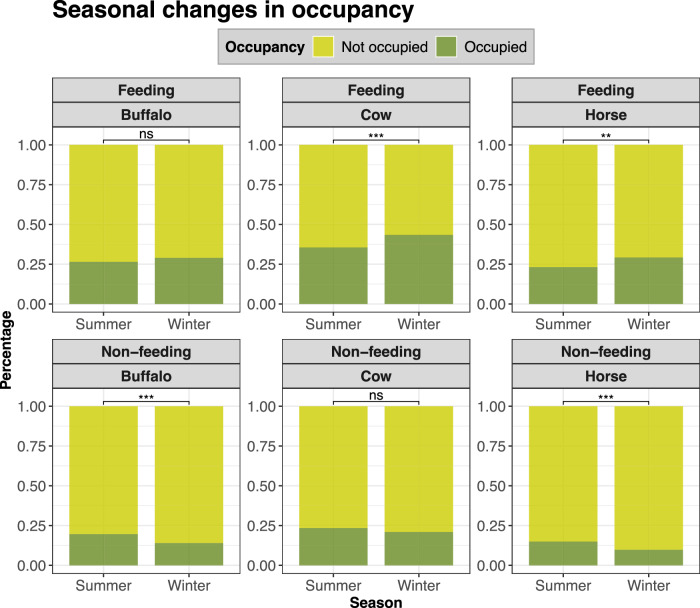
Proportion of occupied (i.e., **>**0 observations) to non-occupied (i.e., **=**0 observations) grid cells, grouped by species and behaviour and comparing summer to winter distribution from the whole study period (4 years). The lines above the bars indicate the results of proportion tests, where one or more “*” indicate a significant difference between the proportions (Z-tests) of occupied to non-occupied grid cells in summer and winter

In addition to the effects of former land use, there were significant seasonal changes in utilized area (i.e., occupied vs. non-occupied grid cells). In winter, a larger part of the study area was used for feeding, while less area was used for non-feeding behaviour compared to summer. These patterns are apparent for all three species, and mostly significant (Fig. 4).

## Discussion

Our study shows that food and water availability, human infrastructure, and former land use shape the habitat selection of large herbivores in this rewilding area, which in turn may alter some of these very qualities (e.g., productivity or vegetation structure). All species show similar seasonal variations: increased ranges for feeding and retracted ranges for non-feeding behaviour in the less productive winter season. Importantly, the impact and importance of several habitat characteristics differs between species (e.g., EVI and proximity to water have little effect on horses compared to buffalo and cows). The interspecific variation in habitat use highlight the importance of functionally diverse herbivore guilds. Overall, these findings mostly substantiate our hypotheses that water, food, former land use and seasonality influence large herbivores. However, we were neither expecting the effects of human infrastructure nor the variation between species (e.g., in their response to EVI).

### Habitat structure and former land use

Wetlands are avoided by all species, which is likely associated with unstable, wet ground and dense vegetation impeding movement and possibly low food quality (high C/N ratios and secondary plant compounds). The comparatively lower visibility and accessibility of wetlands likely decrease detection probability, but the consecutive observation would have documented if animals moved into this habitat type. Thus, we are confident in species’ avoidance of wetlands that our results suggest. Former meadows and pastures offer established, palatable plant communities and increasing plant diversity with age (Waesch and Becker 2009), explaining over-proportional utilization. Moreover, several water bodies, including the artificial pond are found in meadows. A deficit of water bodies - possibly due to anthropogenic drainage for farming – and patchy unpalatable vegetation could explain the lower attractiveness of former arable land. This suggests that the ecological memory of a system (here habitat types and former land use) impacts large-herbivore space use and thus trophic rewilding efforts (Schweiger et al. 2019). So, as hypothesized, land use legacy modulated habitat structure and thus selection of it.

The seasonal changes in used area could be explained by three mechanisms. Firstly, the large herbivores spread out in winter to feed in areas and on resources that are presumably not favourable in summer - especially because all three species show a similar pattern. Secondly, the reduced space use for non-feeding behaviour in winter could reflect tight grouping and clustering in locations that offer protection against harsh weather, including the shelter which appears more attractive in the winter period. Thirdly, wet areas, while important for wallowing, drinking, resting and feeding in summer, are likely less attractive and necessary in winter, when animals try to retain heat, and insects – otherwise deterred by wallowing - are absent. Thus, animals may rather avoid water bodies in winter. Seasonal changes in space are documented for other feral and wild equid population, but the direction of change (e.g., increase or decrease in range size or preferred habitat) appears to be highly system-specific (Schoenecker et al. 2023; St-Louis and Côté 2012). The attraction to water in summer and the range expansion in winter confirmed our hypothesis that habitat selection varies seasonally.

### Water availability

In summer, large herbivores often selected for proximity to water, reflecting drinking and wallowing opportunities. The unattractiveness of natural water bodies compared to the artificial pond, could be partly explained by several water bodies being located in the unfavourable wetlands. Additionally, the artificial pond features gradual slopes, open vegetation and sufficient size and depth to provide drinking and wallowing year-round, while other water bodies are partly overgrown and steep former peat digs. In general, our hypothesis that water access modulates habitat selection is confirmed.

The buffalo preferences for water and wetter parts of the area clearly document the species’ strong association with wet areas. In several rewilding projects in Germany, water buffalos utilize bogs, swamps and floodplains more than other species, supressing shrub encroachment and support birds and amphibians (Wiegleb and Krawczynski 2010).

Being hindgut fermenters horses can retain less water in digestion and are thus more dependent on external water than ruminants (Alexander 1993). Still, they showed a weaker association with water bodies than the two bovids. Not needing to rest and ruminate horses likely move further in between resources and water is abundant in the study area. Thus, while not a consistent predictor in our study, water availability is still to be considered a key factor for equid space use, as has also been shown for other feral horses (Schoenecker et al. 2023).

Overall, TWI showed no clear pattern. Significant negative effects could be attributed to the avoidance of very wet areas, as they offer little resources in a comparatively less accessible terrain, or to the preference of the artificial pond which does not adhere to the topographically produced water distribution.

### Enhanced Vegetation Index (EVI)

Both bovid species selected for higher EVI. This is consistent with former work showing other large herbivores like mule deer or tule elk (*Odocoileus hemionus* and *Cervus canadensis nannodes*) to exhibit similar preferences for vegetation greenness (Hughey et al. 2021; Lendrum et al. 2014). The apparent avoidance of greener areas by horses could reflect smaller-scale selection of certain plants or their parts (e.g., nutritious regrowth) which does not correspond with EVI. Additionally, horses may maintain lawns, that, albeit their productivity, accumulate little aboveground biomass, which would be reflected in EVI (Ödberg and Francis-Smith 1976). Other equids, however, have been shown to seasonally select for NDVI (Giotto et al. 2015). Lower mean EVI in winter compared to summer (0.33 and 0.76 respectively) likely indicates decreased food availability in the colder season supporting the proposed utilization of less favourable resources in winter. Overall, this study supports EVI as a proxy for “vegetation biomass and quality” and thus an important but often system-specific predictor for herbivore space use (Garroutte et al. 2016). Considering the different responses of bovids and horses, our hypothesis that large herbivores select for green biomass was only partially confirmed.

### Infrastructure - fence line, shelter, walking path

The avoidance of the fence by all species could result in an effectively smaller used area, especially at corners and constrictions. However, grid cells close to the fence line have inherently limited access, lowering the probability of random visits, too. Positive effects of the distance to the shelter in summer do not necessarily reflect avoidance but rather preference for former meadows and watering opportunities, which are by chance further away from the shelter. Additionally, daylight is a prerequisite for the visual observations used here, which could mask the role of the shelter for night-time resting. The winter attractiveness of the shelter is likely associated with harsher weather and possibly amplified by the structure’s location in former arable lands, which are used more in winter when the animals are forced to feed in areas and on resources they forgo during the vegetation period. Distance to the path shows mostly non-significant or negative effects. The latter reflect selection for proximity to the path, which may indicate its function for movement, aligning with other studies (Kays et al. 2017). If the path is regarded as a proxy for human activities (e.g., dog-walking, biking, hiking), all three species of large herbivores in our study area appear to be mostly unaffected by current activity levels (see also Knight and Cole 1995). Still, the significant effects especially of the fence lead to the rejection of our fourth hypothesis that infrastructure is not an important predictor due to low use levels and the animals’ familiarity with humans.

### Complementary habitat selection and ecosystem dynamics

This study demonstrates evidence for both niche overlap and niche partitioning for three large herbivores, in line with other work showing resource partitioning in large herbivore assemblages (Mata et al. 2024). Water buffalos and cattle appear more attracted to water than horses and only the ruminants show an association with higher EVI. This aligns with the digestive differences and other studies that describe partly overlapping but differing ecological niches of bovids (ruminants) and horses (hindguts) (e.g., Cromsigt et al. 2018; Vulink et al. 2001; Zielke et al. 2019; Popp and Scheibe 2014). The differences between bovids and horses often extend to their respective effects on vegetation structure - e.g. regulation of woody vegetation by the former and maintenance of grazing lawns by the latter (Linnartz and Meissner 2014; Lamoot et al. 2005). The clear association of water buffalos with water reflects the species’ distinct niche and aligns with other evidence of buffalos increasing structural heterogeneity and species richness, especially in wetlands (e.g., Wiegleb and Krawczynski 2010; Gray et al. 2019; see also Lundgren et al. 2018). Overall, horses, cattle and water buffalos complement each other in their space use and thus likely also in their ecological impacts and ecosystem engineering. Therefore, we recommend that rewilding should always thrive for a diverse herbivore assemblage (Svenning et al. 2024b).

**Fig. 5:**
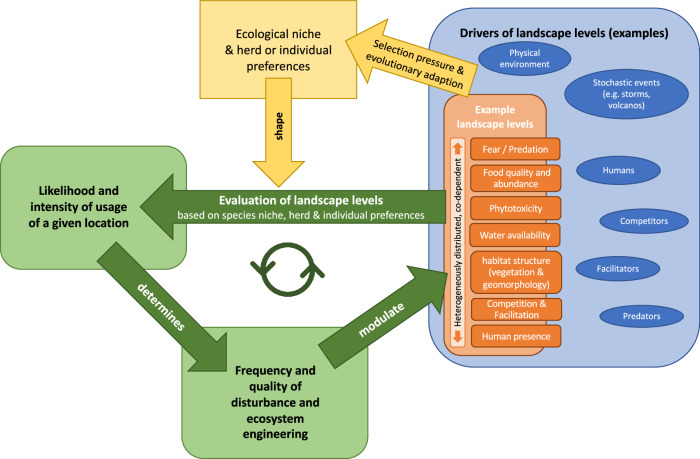
Conceptual model of ecosystem dynamics and self-regulation by herbivores. Different landscape levels, such as food and water availability or predation risk (orange), are evaluated by animals reflecting their ecological niche, herd, and individual preferences (yellow). For example, large herbivores perceive lower predation risks and different food availability than small ones. Through the resulting habitat selection, animals also modulate where and in which intensity their disturbance and habitat engineering occur (green). So, herbivores modulate some of the landscape levels on which they base their own habitat selection, thereby partly self-regulating in feedback (green). Additional drivers of landscape levels (e.g., stochastic events) are named (blue)

We demonstrate that large-herbivore habitat selection is based on several habitat qualities, including food availability, water bodies, land use legacy, and human infrastructure. Other studies identify additional elements influencing large herbivores, including vegetation structure, phytotoxicity or fear (e.g., Hunter 2016; Gaynor et al. 2019). As all these factors are heterogeneously distributed in space and time, so are the large herbivores and the multitude of ecological impacts (e.g., biomass removal, excrements) they have (Trepel et al. 2024; Svenning et al. 2019). Through these impacts large herbivores influence the qualities they base their habitat selection on (e.g., modulating wildfire regimes and feeding on post-fire regrowth (Johnson et al. 2018; Archibald et al. 2005)). Large herbivores also shape the space use of other species (e.g., African elephants modify vegetation structure, indirectly changing the landscape of fear (Fležar et al. 2019)). Additionally, habitat selection is individual, herd- and habitat-specific and often departs from assumed ecological niches (e.g., Kuemmerle et al. 2018). Many landscape characteristics are not nested hierarchically but rather interact with each other and are partly shaped by large herbivores. We propose a dynamic feedback-system, where herbivores partly self-regulate by shaping the very same landscape qualities (e.g., plant productivity) they base their habitat selection on (Fig. 5).

Seasonal, behavioural, inter-species, inter-group and individual variability in habitat selection should be expected to generate a similar variability in the intensity and frequency of disturbance and ecosystem engineering in space and time, which in turn would promote habitat heterogeneity and biodiversity. Full self-sufficiency of such a system would rely on large-scale rewilding efforts that include diverse herbivore and carnivore guilds and long-distance migration (e.g., Svenning et al. 2016; Sinclair et al. 2007).

### Rewilding management

We found anthropogenic habitat variability (e.g., land use legacy, water management) shaping large-herbivore habitat selection. For example, while intended to prevent disturbance of amphibians in other water bodies, the artificial pond also profoundly impacts animal space use (Aarhus municipality, personal communication) and hence their ecological effects. Rarely used areas (e.g., peatlands) may serve as refuges for species sensitive to large herbivore disturbance. So, environmental variability (e.g., hydrology, topography) and anthropogenic habitat variability affect habitat selection and need to be considered, especially if newly introduced through the project (e.g., fences, shelter). This is especially important in highly modified landscapes and small projects, where rewilding gradually fades into human management (Jepson and Schepers 2016).

We found seasonal range and behaviour shifts that indicate adapted resource use (i.e., feeding on lower quality feed in winter). Enabling migration in response to changing resources could decrease the need for management, inbreeding and stochastic risks. If large-scale rewilding is implemented migrating herbivores could increase biotic connectivity and possibly support the range shifts, that many species exhibit in adaption to global change (Berti and Svenning 2020; Perino et al. 2019; see also Lenoir and Svenning 2015). Holistic assessment of rewilding potential and subsequent political prioritization should evaluate existing bio- and geodiversity, disturbances, area size and habitat connectivity (Bergin et al. 2024).

### Limitations

The choice of animal groups, effort, location approximation, and timing of observations were potentially suboptimal. There is risk that (subconscious) convenience attracts observers to established paths, generating unequal sampling. Oversampling of certain animal groups and characteristic locations (e.g., artificial pond) is likely. Information of no animal encounter (true zero) is lost. Locations are approximated and data is biased towards daytime and possibly also towards good weather. Lack of nighttime observations has been linked to distorted habitat selection patterns (Beyer Jr and Haufler 1994). The absence of consecutive time series limited this study to spatial patterns, but temporal variability is an important dimension of habitat selection (e.g., Dupke et al. 2017). Lastly, as the animals have different herd sizes and social structures (~40 cattle of both sexes compared to 3 male water buffalos and ~6 horses) representativeness for the species is weakened. Albeit these weaknesses, our data and especially the recorded behaviour yields interesting insights and can be considered dependable, as the space use patterns, we identify, align with unpublished work on vegetation, plant biomass and dung counts.

### Outlook

Our results show that human infrastructure (paths, fences, shelters), watering opportunities (especially the artificial one) and food quantity (proxied by EVI) shape the habitat selection of large herbivores in our study area. Former meadows were preferred over former arable land, rendering prior land use and thus ecological memory an important determinant of space use in line with Schweiger et al. (2019). Within the study site, wetlands are poorly accessible for large herbivores, offer little resources, and were thus avoided. Habitat preferences shifted seasonally, resulting in a heterogeneous spatiotemporal distribution of the large grazers and likely also their impacts. Further, horses, cattle, and water buffalos had differing habitat preferences. The species complement each other in their space use and thus likely also in their ecological impacts, underlining the importance of diversified herbivore guilds. So, the diversity of large consumers, area size, habitat connectivity, existing bio- and geodiversity and natural and anthropogenic disturbances shape rewilding potential, system functioning and resilience.

Habitat selection of other species, in other ecoregions and additional habitat qualities (e.g., plant toxicity, vegetation structure, predator fear effects) demand more research. Furthermore, self-regulation of space use and the influence of large and megaherbivores on nutrient and water cycles, succession and alternative ecosystem states in rewilding contexts in Europe and elsewhere should be further investigated, also to inform implementation of rewilding actions.

Nature is intrinsically ever evolving and dynamic. As large herbivores are important drivers of this eternal motion, science and practice should embrace their importance and potential much more.

## Supplementary information

Supplementary material

## Data Availability

Data can be made available upon request. We aim for full transparency.

## References

[CR1] Alexander RM (1993) The relative merits of foregut and hindgut fermentation. J Zool 231:391–401

[CR2] Archibald S, Bond W, Stock W, Fairbanks D (2005) Shaping the landscape: fire–grazer interactions in an African savanna. Ecol applic 15:96–109

[CR3] Assmann JJ, Moeslund JE, Treier UA, Normand S (2022) EcoDes-DK15: high-resolution ecological descriptors of vegetation and terrain derived from Denmark’s national airborne laser scanning data set. Earth Syst Sci Data 14:823–844. 10.5194/essd-14-823-2022

[CR4] Bergin MD, Pedersen RØ, Jensen M, Svenning J-C (2024) Mapping rewilding potential – A systematic approach to prioritise areas for rewilding in human-dominated regions. J Nat Conserv 77:126536. 10.1016/j.jnc.2023.126536

[CR5] Bergman J, Pedersen RØ, Lundgren EJ, Lemoine RT, Monsarrat S, Pearce EA, Schierup MH, Svenning J-C (2023) Worldwide Late Pleistocene and Early Holocene population declines in extant megafauna are associated with Homo sapiens expansion rather than climate change. Nat Commun 14: 7679. 10.1038/s41467-023-43426-5 37996436 10.1038/s41467-023-43426-5 PMC10667484

[CR6] Berti E, Svenning J-C (2020) Megafauna extinctions have reduced biotic connectivity worldwide. Glob Ecol Biogeogr 29:2131–2142. 10.1111/geb.13182

[CR7] Beyer Jr E, Haufler JB (1994) Diurnal versus 24-hour sampling of habitat use. J Wildl Manag 27:178–180

[CR8] Blicharska M, Smithers RJ, Mikusiński G, Rönnbäck P, Harrison PA, Nilsson M, Sutherland WJ (2019) Biodiversity’s contributions to sustainable development. Nat Sustain 2:1083–1093. 10.1038/s41893-019-0417-9

[CR9] Bocherens H (2018) The rise of the anthroposphere since 50,000 years: an ecological replacement of megaherbivores by humans in terrestrial ecosystems?. F Ecol Evo 6:3. 10.3389/fevo.2018.00003

[CR10] Brooks ME, Kristensen K, van, Benthem KJ, Magnusson A, Berg CW, Nielsen A, Skaug HJ, Machler M, Bolker BM (2017) glmmTMB balances speed and flexibility among packages for zero-inflated generalized linear mixed modeling. R J 9:378–400. 10.32614/RJ-2017-066

[CR11] Burkepile DE, Burns CE, Tambling CJ, Amendola E, Buis GM, Govender N, Nelson V, Thompson DI, Zinn AD, Smith MD (2013) Habitat selection by large herbivores in a southern African savanna: the relative roles of bottom-up and top-down forces. Ecosphere 4:art139. 10.1890/ES13-00078

[CR12] Burnham KP, Anderson DR (2004) Multimodel inference: understanding AIC and BIC in model selection. Sociol Methods Res 33:261–304. 10.1177/0049124104268644

[CR13] Butchart SHM, Walpole M, Collen B, van Strien A, Scharlemann JPW, Almond REA, Baillie JEM, Bomhard B, Brown C, Bruno J, Carpenter KE, Carr GM, Chanson J, Chenery AM, Csirke J, Davidson NC, Dentener F, Foster M, Galli A, Galloway JN, Genovesi P, Gregory RD, Hockings M, Kapos V, Lamarque J-F, Leverington F, Loh J, McGeoch MA, McRae L, Minasyan A, Morcillo MH, Oldfield TEE, Pauly D, Quader S, Revenga C, Sauer JR, Skolnik B, Spear D, Stanwell-Smith D, Stuart SN, Symes A, Tierney M, Tyrrell TD, Vié J-C, Watson R (2010) Global Biodiversity: Indicators of Recent Declines. Science 328:1164–1168. 10.1126/science.1187512 20430971 10.1126/science.1187512

[CR14] Büttner G (2014) CORINE Land Cover and Land Cover Change Products. In: Manakos I, Braun M (eds) Land Use and Land Cover Mapping in Europe. Remote Sensing and Digital Image Processing, vol 18. Springer, 10.1007/978-94-007-7969-3_5

[CR15] Büttner G, Maucha G (2006) The thematic accuracy of Corine land cover 2000 Assessment using LUCAS (land use/cover area frame statistical survey), EEA Technical report No 7/2006. European Environment Agency (EEA) Copenhagen. Retrieved from: https://land.copernicus.eu/api/en/technical-library/report-on-clc-2000-thematic-accuracy-assessment-using-lucas. Last accessed 05.02.2025

[CR16] Cardinale BJ, Duffy JE, Gonzalez A, Hooper DU, Perrings C, Venail P, Narwani A, Mace GM, Tilman D, Wardle DA, Kinzig AP, Daily GC, Loreau M, Grace JB, Larigauderie A, Srivastava DS, Naeem S (2012) Biodiversity loss and its impact on humanity. Nature 486:59–67. 10.1038/nature11148 22678280 10.1038/nature11148

[CR17] Chapin III FS, Zavaleta ES, Eviner VT, Naylor RL, Vitousek PM, Reynolds HL, Hooper DU, Lavorel S, Sala OE, Hobbie SE, Mack MC, Díaz S (2000) Consequences of changing biodiversity. Nature 405:234–242. 10.1038/35012241 10821284 10.1038/35012241

[CR18] Cowie RH, Bouchet P, Fontaine B (2022) The Sixth Mass Extinction: fact, fiction or speculation?. Biol Rev 97:640–663. 10.1111/brv.12816 35014169 10.1111/brv.12816 PMC9786292

[CR19] Cromsigt JP, Kemp YJ, Rodriguez E, Kivit H (2018) Rewilding Europe’s large grazer community: how functionally diverse are the diets of European bison, cattle, and horses?. Restor Ecol 26:891–899. 10.1111/rec.12661

[CR20] Danish Agency for Data Supply and Infrastructure (2021) Orthophoto 10cm resolution, https://datafordeler.dk/dataoversigt/. Accessed 05.02.2025

[CR21] Davoli M, Monsarrat S, Pedersen RØ, Scussolini P, Karger DN, Normand S, Svenning J-C (2024) Megafauna diversity and functional declines in Europe from the Last Interglacial to the present. Glob Ecol Biogeogr 33:34–47. 10.1101/2023.06.12.544580

[CR22] Dupke C, Bonenfant C, Reineking B, Hable R, Zeppenfeld T, Ewald M, Heurich M (2017) Habitat selection by a large herbivore at multiple spatial and temporal scales is primarily governed by food resources. Ecography 40:1014–1027. 10.1111/ecog.02152

[CR23] Earth Engine Data Catalog (2022) Harmonized Sentinel-2 MSI: MultiSpectral Instrument, Level-2A, https://developers.google.com/earth-engine/datasets/catalog/COPERNICUS_S2_SR_HARMONIZED#description. Acessed 05.02.2024

[CR24] Enquist BJ, Abraham AJ, Harfoot MBJ, Malhi Y, Doughty CE (2020) The megabiota are disproportionately important for biosphere functioning. Nat Commun 11:699. 10.1038/s41467-020-14369-y 32019918 10.1038/s41467-020-14369-y PMC7000713

[CR25] European Environmental Agency (EEA) (2012) CORINE Land Cover 2012 (vector/raster 100 m), Europe, 6-yearly, https://land.copernicus.eu/en/products/corine-land-cover/clc-2012. Accessed 05.02.2025

[CR26] Fležar U, Le Roux E, Kerley GIH, Kuijper DPJ, te Beest M, Druce DJ, Prinsloo D, Cromsigt JPGM (2019) Simulated elephant-induced habitat changes can create dynamic landscapes of fear. Biol Conserv 237:267–279. 10.1016/j.biocon.2019.07.012

[CR27] Garroutte EL, Hansen AJ, Lawrence RL (2016) Using NDVI and EVI to Map Spatiotemporal Variation in the Biomass and Quality of Forage for Migratory Elk in the Greater Yellowstone Ecosystem. Remote Sens 8:404. 10.3390/rs8050404

[CR28] Gaynor KM, Brown JS, Middleton AD, Power ME, Brashares JS (2019) Landscapes of Fear: Spatial Patterns of Risk Perception and Response. Trends Ecol Evol 34:355–368. 10.1016/j.tree.2019.01.004 30745252 10.1016/j.tree.2019.01.004

[CR29] Giotto N, Gerard J-F, Ziv A, Bouskila A, Bar-David S (2015) Space-use patterns of the Asiatic wild ass (Equus hemionus): complementary insights from displacement, recursion movement and habitat selection analyses. PLoS One 10:e0143279. 10.1371/journal.pone.0143279 26630393 10.1371/journal.pone.0143279 PMC4667895

[CR30] Gorelick N, Hanscher M, Dixon M, Ilyushchenko S, Thau D, Moore R et al. (2017) Google Earth Engine: Planetary-scale geospatial analysis for everyone. Remote Sens Environ 202:18–27. 10.1016/j.rse.2017.06.031

[CR31] Gray T, Eames J, Lyon J, Meyerhoff M (2019) Rewilding in Southeast Asia: an assessment of conservation. Camb J Nat Hist 2019:98–112

[CR32] Hartig F (2022) DHARMa: Residual Diagnostics for Hierarchical (Multi-Level / Mixed) Regression Models. R package version 0.4.6, http://florianhartig.github.io/DHARMa/. Acessed 05.02.2025

[CR33] Huete A, Didan K, Miura T, Rodriguez EP, Gao X, Ferreira LG (2002) Overview of the radiometric and biophysical performance of the MODIS vegetation indices. Remote Sens Environ 83:195–213. 10.1016/S0034-4257(02)00096-2

[CR34] Hughey LF, Shoemaker KT, Stewart KM, McCauley DJ, Cushman JH (2021) Effects of human-altered landscapes on a reintroduced ungulate: Patterns of habitat selection at the rangeland-wildland interface. Biol Conserv 257:109086. 10.1016/j.biocon.2021.109086

[CR35] Hunter MD (2016) The Phytochemical Landscape. Princeton, Princeton University Press, 10.1515/9781400881208

[CR36] Jepson P, Schepers F (2016) Making space for rewilding: creating an enabling policy environment. Rewilding Europe, https://rewildingeurope.com/wp-content/uploads/2016/05/Making-Space-for-Rewilding-Policy-Brief1.pdf. Accessed 05.02.2025

[CR37] Johnson CN, Prior LD, Archibald S, Poulos HM, Barton AM, Williamson GJ, Bowman DM (2018) Can trophic rewilding reduce the impact of fire in a more flammable world?. Philos Trans R Soc B 373:20170443. 10.1098/rstb.2017.0443 10.1098/rstb.2017.0443 PMC6231065 30348870

[CR38] Kays R, Parsons AW, Baker MC, Kalies EL, Forrester T, Costello R, Rota CT, Millspaugh JJ, McShea WJ (2017) Does hunting or hiking affect wildlife communities in protected areas?. J Appl Ecol 54:242–252. 10.1111/1365-2664.12700

[CR39] Knight RL, Cole DN (1995) Wildlife responses to recreationists. In: Knight RL, Gutzwiller KJ (eds) Wildlife and recreationists: Coexistence through management and research. Island Press, Washington, DC, pp 51–69

[CR40] Kuemmerle T, Levers C, Bleyhl B, Olech W, Perzanowski K, Reusch C, Kramer-Schadt S (2018) One size does not fit all: European bison habitat selection across herds and spatial scales. Landsc Ecol 33:1559–1572. 10.1007/s10980-018-0684-2

[CR41] Lamoot I, Meert C, Hoffmann M (2005) Habitat use of ponies and cattle foraging together in a coastal dune area. Biol Conserv 122:523–536. 10.1016/j.biocon.2004.09.007

[CR42] Lendrum PE, Anderson CR, Monteith KL, Jenks JA, Bowyer RT (2014) Relating the movement of a rapidly migrating ungulate to spatiotemporal patterns of forage quality. Mamm Biol 79:369–375. 10.1016/j.mambio.2014.05.005

[CR43] Lenoir J, Svenning J-C (2015) Climate-related range shifts – a global multidimensional synthesis and new research directions. Ecography 38:15–28. 10.1111/ecog.00967

[CR44] Linnartz L, Meissner R (2014) Rewilding horses in Europe: background and guidelines-a living document. Rewilding Europe, https://www.rewildingeurope.com/wp-content/uploads/2014/09/Rewilding-horses-in-Europe-2014.pdf. Acessed 05.02.2025

[CR45] Liu HQ, Huete A (1995) A feedback based modification of the NDVI to minimize canopy background and atmospheric noise. IEEE Trans Geosci Remote Sens 33:457–465. 10.1109/TGRS.1995.8746027

[CR46] Lundgren EJ, Bergman J, Trepel J, Le Roux E, Monsarrat S, Kristensen JA, Pedersen RØ, Pereyra P, Tietje M, Svenning J-C (2024) Functional traits—not nativeness—shape the effects of large mammalian herbivores on plant communities. Science 383:531–537. 10.1126/science.adh2616 38301018 10.1126/science.adh2616

[CR47] Lundgren EJ, Ramp D, Ripple WJ, Wallach AD (2018) Introduced megafauna are rewilding the Anthropocene. Ecography 41:857–866. 10.1111/ecog.03430

[CR48] Malhi Y, Lander T, Le Roux E, Stevens N, Macias-Fauria M, Wedding L, Girardin C, Kristensen JÅ, Sandom CJ, Evans TD (2022) The role of large wild animals in climate change mitigation and adaptation. Curr Biol 32:R181–R196. 10.1016/j.cub.2022.01.041 35231416 10.1016/j.cub.2022.01.041

[CR49] Mata JC, Davison CW, Frøslev TG, Buitenwerf R, Svenning JC (2024) Resource partitioning in a novel herbivore assemblage in South America. J Anim Ecol 93:606–618. 10.1111/1365-2656.14069 38414265 10.1111/1365-2656.14069

[CR50] Mazor T, Doropoulos C, Schwarzmueller F, Gladish DW, Kumaran N, Merkel K, Di Marco M, Gagic V (2018) Global mismatch of policy and research on drivers of biodiversity loss. Nat Ecol Evol 2:1071–1074. 10.1038/s41559-018-0563-x 29784980 10.1038/s41559-018-0563-x

[CR51] Müller A, Dahm M, Bøcher PK, Root-Bernstein M, Svenning J-C (2017) Large herbivores in novel ecosystems-Habitat selection by red deer (Cervus elaphus) in a former brown-coal mining area. PLoS One 12:e0177431. 10.1371/journal.pone.0177431 28505192 10.1371/journal.pone.0177431 PMC5432106

[CR52] Ödberg FO, Francis-Smith K (1976) A Study on Eliminative and Grazing Behaviour — The Use of the Field by Captive Horses. Equine Vet J 8:147–149. 10.1111/j.2042-3306.1976.tb03326.x

[CR53] Pedersen PBM, Ejrnæs R, Sandel B, Svenning J-C (2020) Trophic Rewilding Advancement in Anthropogenically Impacted Landscapes (TRAAIL): A framework to link conventional conservation management and rewilding. Ambio 49:231–244. 10.1007/s13280-019-01192-z 31201614 10.1007/s13280-019-01192-z PMC6889113

[CR54] Perino A, Pereira HM, Navarro LM, Fernández N, Bullock JM, Ceaușu S, Cortés-Avizanda A, van Klink R, Kuemmerle T, Lomba A, Pe’er G, Plieninger T, Rey Benayas JM, Sandom CJ, Svenning J-C, Wheeler HC (2019) Rewilding complex ecosystems. Science 364:eaav5570. 10.1126/science.aav5570 31023897 10.1126/science.aav5570

[CR55] Pires MM, Guimarães PR, Galetti M, Jordano P (2018) Pleistocene megafaunal extinctions and the functional loss of long-distance seed-dispersal services. Ecography 41:153–163. 10.1111/ecog.03163

[CR56] Popp A, Scheibe KM (2014) The ecological influence of large herbivores–behavior and habitat utilization of cattle and horses. Appl Ecol Environ Res 12:681–693. 10.15666/aeer/1203_681693

[CR57] Ripple WJ, Newsome TM, Wolf C, Dirzo R, Everatt KT, Galetti M, Hayward MW, Kerley GI, Levi T, Lindsey PA (2015) Collapse of the world’s largest herbivores. Sci Adv 1:e1400103. 10.1126/sciadv.1400103 26601172 10.1126/sciadv.1400103 PMC4640652

[CR58] Sandom C, Faurby S, Sandel B, Svenning J-C (2014) Global late Quaternary megafauna extinctions linked to humans, not climate change. Proc R Soc B 281:20133254. 10.1098/rspb.2013.3254 10.1098/rspb.2013.3254 PMC4071532 24898370

[CR59] Schmitz OJ, Sylvén M, Atwood TB, Bakker ES, Berzaghi F, Brodie JF, Cromsigt JPGM, Davies AB, Leroux SJ, Schepers FJ, Smith FA, Stark S, Svenning J-C, Tilker A, Ylänne H (2023) Trophic rewilding can expand natural climate solutions. Nat Clim Chang 13:324–333. 10.1038/s41558-023-01631-6

[CR60] Schoenecker KA, Esmaeili S, King SRB (2023) Seasonal resource selection and movement ecology of free-ranging horses in the western United States. J Wildl Manag 87:e22341. 10.1002/jwmg.22341

[CR61] Schweiger AH, Boulangeat I, Conradi T, Davis M, Svenning J-C (2019) The importance of ecological memory for trophic rewilding as an ecosystem restoration approach. Biol Rev 94:1–15. 10.1111/brv.12432 29877019 10.1111/brv.12432

[CR62] Sinclair ARE, Mduma SAR, Hopcraft JGC, Fryxell JM, Hilborn R, Thirgood S (2007) Long-Term Ecosystem Dynamics in the Serengeti: Lessons for Conservation. Conserv Biol 21:580–590. 10.1111/j.1523-1739.2007.00699.x 17531037 10.1111/j.1523-1739.2007.00699.x

[CR63] Søndergaard SA, Fløjgaard C, Ejrnæs R, Svenning J-C (2025) Shifting baselines and the forgotten giants: integrating megafauna into plant community ecology. Oikos e11134. 10.1111/oik.11134

[CR64] St-Louis A, Côté SD (2012) Foraging behaviour at multiple temporal scales in a wild alpine equid. Oecologia 169:167–176. 10.1007/s00442-011-2166-y 22033764 10.1007/s00442-011-2166-y

[CR65] Svenning J-C, Lemoine RT, Bergman J, Buitenwerf R, Le Roux E, Lundgren E, Mungi N, Pedersen RØ (2024a) The late-Quaternary megafauna extinctions: Patterns, causes, ecological consequences and implications for ecosystem management in the Anthropocene. Camb Prisms Extinct 2:e5. 10.1017/ext.2024 10.1017/ext.2024.4 PMC11895740 40078803

[CR66] Svenning J-C, Buitenwerf R, Le Roux E (2024b) Trophic rewilding as a restoration approach under emerging novel biosphere conditions. Curr Biol 34:R435–R451. 10.1016/j.cub.2024.02.044 38714176 10.1016/j.cub.2024.02.044

[CR67] Svenning J-C, Munk M, Schweiger A (2019) Trophic rewilding: ecological restoration of top-down trophic interactions to promote self-regulating biodiverse ecosystems. In: Perino A, Pereira HM (eds) Rewilding. Cambridge University Press, pp 73–89. 10.1017/9781108560962.005

[CR68] Svenning J-C, Pedersen PBM, Donlan CJ, Ejrnaes R, Faurby S, Galetti M, Hansen DM, Sandel B, Sandom CJ, Terborgh JW, Vera FWM (2016) Science for a wilder Anthropocene: Synthesis and future directions for trophic rewilding research. Proc Natl Acad Sci USA 113:898–906. 10.1073/pnas.1502556112 26504218 10.1073/pnas.1502556112 PMC4743824

[CR69] Taylor B, Chapron G, Kopnina H, Orlikowska E, Gray J, Piccolo JJ (2020) The need for ecocentrism in biodiversity conservation. Conserv Biol 34:1089–1096. 10.1111/cobi.13541 32424955 10.1111/cobi.13541

[CR70] Trepel J, Le Roux E, Abraham AJ, Buitenwerf R, Kamp J, Kristensen JA, Tietje M, Lundgren EJ, Svenning J-C (2024) Meta-analysis shows that wild large herbivores shape ecosystem properties and promote spatial heterogeneity. Nat Ecol Evol, 10.1038/s41559-024-02327-6 10.1038/s41559-024-02327-6 38337048

[CR71] V. 3.24 Tisler; QGIS development team (2022) QGIS Geographic Information System, https://qgis.org/. Accessed 05.02.2025

[CR72] V. 4.2.1; R Core Team (2022) R: A language and environment for statistical computing. R Foundation for Statistical Computing, Vienna, Austria, https://www.R-project.org/. Acessed 05.02.2025

[CR73] Vulink J, Cornelissen P, Drost H, Prins H (2001) Hindgut fermentation is not an evolutionary dead end: comparative feeding ecology of cattle and horses. In: Vulink J (ed) Hungry herds: management of temperate lowland wetlands by grazing, Van Zee tot Land, No 66, pp 87–103

[CR74] Waesch G, Becker T (2009) Plant diversity differs between young and old mesic meadows in a central European low mountain region. Agric Ecosyst Environ 129:457–464. 10.1016/j.agee.2008.10.022

[CR75] Wang Y, Liu Q (2006) Comparison of Akaike information criterion (AIC) and Bayesian information criterion (BIC) in selection of stock–recruitment relationships. Fish Res 77:220–225. 10.1016/j.fishres.2005.08.011

[CR76] Wiegleb G, Krawczynski R (2010) Biodiversity management by water buffalos in restored wetlands. Waldökologie Landschaftsforschung Naturschutz 10:17–22

[CR77] Xu H, Cao Y, Yu D, Cao M, He Y, Gill M, Pereira HM (2021) Ensuring effective implementation of the post-2020 global biodiversity targets. Nat Ecol Evol 5:411–418. 10.1038/s41559-020-01375-y 33495589 10.1038/s41559-020-01375-y

[CR78] Zielke L, Wrage-Mönnig N, Müller J, Neumann C (2019) Implications of spatial habitat diversity on diet selection of European Bison and Przewalski’s Horses in a Rewilding Area. Diversity 11:63. 10.3390/d11040063

