# Supplementary material for: What moves large grazers? Habitat preferences and complementing niches of large herbivores in a Danish trophic rewilding area
Source: Environ Manage. 2025 Apr 25;75(7):1665–79. doi: 10.1007/s00267-025-02164-8 (PMC12228648; doi:10.1007/s00267-025-02164-8)
Supplement: Supplementary file 1 — Supplementary material [file 267_2025_2164_MOESM1_ESM.docx]

**Supplementary material**


**Supplementary Figure 1:** Collection of representative images of (A) cattle, (B) water buffalo, (C) horses, (D) the man-made shelter, (E) the fence line, (F1 & F2) the artificial pond, (G) the walking paths, (H) former peat- and wetlands, (I) former meadows, (J) former arable land

**Supplementary Figure 2: Residuals for models of cattle.** The QQ-plots on the left show observed (y-axis) versus expected (x-axis) values, while the plots on the right compare the simulated residuals (y-axis) with the model predictions (x-axis). Stars indicate outliers. Plot A is based on the model of feeding cattle in the summer, B on non-feeding cattle in the summer, C on feeding cattle in the winter and D on non-feeding cattle in the winter.

**Supplementary Figure 3: Residuals for models of horses.** The QQ-plots on the left show observed (y-axis) versus expected (x-axis) values, while the plots on the right compare the simulated residuals (y-axis) with the model predictions (x-axis). Stars indicate outliers. Plot A is based on the model of feeding horses in the summer, B on non-feeding horses in the summer, C on feeding horses in the winter and D on non-feeding horses in the winter.

**Supplementary Figure 4: Residuals for models of buffalos.** The QQ-plots on the left show observed (y-axis) versus expected (x-axis) values, while the plots on the right compare the simulated residuals (y-axis) with the model predictions (x-axis). Stars indicate outliers. Plot A is based on the model of feeding buffalos in the summer, B on non-feeding buffalos in the summer, C on feeding buffalos in the winter and D on non-feeding buffalos in the winter.

**Supplementary Table 1: Observed behaviours described and categorized.** When conducting survey blocks in the field observers would assign observations one of 11 possible codes signifying a certain clearly described behaviour. Ruminating was only initially used as a category as distinguishment from resting appeared challenging. For this study behaviours were later grouped in feeding and non-feeding.

| **Behaviour** | **Description of the behaviour** | **Code during observartion** | **Catergory for modelling** |
| --- | --- | --- | --- |
| Grazing | Eating grass or herbs | 1 | Feeding |
| Browsing | Eating woody plants (leaves, twigs, bark) | 2 | Feeding |
| Standing | Standing still | 3 | Non-feeding |
| Walking | Walking without grazing | 4 | Non-feeding |
| Resting | Sleeping or lying down ~~(but not ruminating)~~ | 5 | Non-feeding |
| ~~Ruminating~~ | ~~For cattle/water buffalo only: ruminating~~ | ~~6~~ | Non-feeding |
| Rubbing | Rubbing/scratching e.g. on trees, fences | 7 | Non-feeding |
| Lactating | Mother giving milk to young | 8 | Non-feeding |
| Drinking | Drinking water | 9 | Non-feeding |
| Wallowing | Wallowing in mud or water | 10 | Non-feeding |
| Other | Anything different from the above | 11 | Non-feeding |

**Supplementary Table 2 Number of observations per year of the study period and split by species**

| **Year** | **No. Observation** | **Species** | **No. Observation** |
| --- | --- | --- | --- |
|  |  | Cattle | 847 |
| **2018** | **2465** | Horse | 798 |
|  |  | Buffalo | 820 |
|  |  | Cattle | 2025 |
| **2019** | **5820** | Horse | 1892 |
|  |  | Buffalo | 1903 |
|  |  | Cattle | 6065 |
| **2020** | **14514** | Horse | 2117 |
|  |  | Buffalo | 6332 |
|  |  | Cattle | 863 |
| **2021** | **2687** | Horse | 854 |
|  |  | Buffalo | 970 |
| **Total** | **25486** |  | **25486** |

**Supplementary Table 3: Model estimates, standard errors, and p values for the GLMMs for cattle.** The first column contains the covariates. Each model is represented in three columns (e.g. model of feeding cattle in the summer is in columns 2,3 & 4). Columns with “EST” show model estimates, columns with “SE” show standard errors and columns with “p” show the p-value of the variable in the given model. P-values are non-significant when p >0.05, indicated by “ns”.

| **Variable** | **Feeding** | | | | | | **Non-feeding** | | | | | |
| --- | --- | --- | --- | --- | --- | --- | --- | --- | --- | --- | --- | --- |
|  | **Summer** | | | **Winter** | | | **Summer** | | | **Winter** | | |
|  | ***EST*** | ***SE*** | ***p*** | ***EST*** | ***SE*** | ***p*** | ***EST*** | ***SE*** | ***p*** | ***EST*** | ***SE*** | ***p*** |
| **Intercept (full)** | -2.917 | 0.653 | <0.001 | -1.857 | 0.714 | <0.01 | -4.259 | 0.618 | <0.001 | -3.481 | 0.671 | <0.001 |
| **EVI** | 0.286 | 0.053 | <0.001 | 0.572 | 0.064 | <0.001 | 0.063 | 0.081 | ns | 0.370 | 0.099 | <0.001 |
| **TWI** | -0.002 | 0.054 | ns | -0.071 | 0.054 | ns | -0.267 | 0.090 | <0.01 | -0.124 | 0.091 | ns |
| **Dist. pond (a.)** | -1.158 | 0.171 | <0.001 | -0.005 | 0.165 | ns | -2.110 | 0.305 | <0.001 | 0.160 | 0.243 | ns |
| **Dist. ponds (n.)** | -0.222 | 0.073 | <0.01 | -0.248 | 0.086 | <0.01 | -0.824 | 0.128 | <0.001 | -0.249 | 0.136 | ns |
| **Dist. path** | -0.042 | 0.144 | ns | -0.128 | 0.151 | ns | 0.921 | 0.236 | <0.001 | 0.345 | 0.239 | ns |
| **Dist. fence** | 0.119 | 0.056 | <0.05 | 0.566 | 0.061 | <0.001 | 0.556 | 0.095 | <0.001 | 0.971 | 0.095 | <0.001 |
| **Intercept (red.)** | -2.785 | 0.704 | <0.001 | -1.716 | 0.709 | <0.05 | -4.048 | 0.786 | <0.001 | -3.351 | 0.722 | <0.001 |
| **Dist. shelter** | 0.148 | 0.106 | ns | -0.316 | 0.116 | <0.01 | -0.319 | 0.179 | ns | -0.671 | 0.170 | <0.001 |

**Supplementary Table 4: Model estimates, standard errors, and p values for the GLMMs for horses.** The first column contains the covariates. Each model is represented in three columns (e.g. model of feeding horses in the summer is in columns 2,3 & 4). Columns with “EST” show model estimates, columns with “SE” show standard errors and columns with “p” show the p-value of the variable in the given model. P-values are non-significant when p >0.05, indicated by “ns”.

| **Variable** | **Feeding** | | | | | | **Non-feeding** | | | | | |
| --- | --- | --- | --- | --- | --- | --- | --- | --- | --- | --- | --- | --- |
|  | **Summer** | | | **Winter** | | | **Summer** | | | **Winter** | | |
|  | **EST** | **SE** | **p** | **EST** | **SE** | **p** | **EST** | **SE** | **p** | **EST** | **SE** | **p** |
| **Intercept (full)** | -4.775 | 1.994 | <0.05 | -2.369 | 0.926 | <0.05 | -5.603 | 1.800 | <0.01 | -4.433 | 0.729 | <0.001 |
| **EVI** | -0.290 | 0.082 | <0.001 | 0.071 | 0.089 | ns | -0.289 | 0.119 | <0.05 | 0.268 | 0.145 | ns |
| **TWI** | 0.129 | 0.081 | ns | -0.107 | 0.077 | ns | -0.400 | 0.123 | <0.01 | -0.169 | 0.144 | ns |
| **Dist. pond (a.)** | -0.418 | 0.224 | ns | 0.740 | 0.204 | <0.001 | -0.928 | 0.324 | <0.01 | 0.558 | 0.314 | ns |
| **Dist. ponds (n.)** | 0.273 | 0.112 | <0.05 | -0.309 | 0.103 | <0.01 | -0.438 | 0.164 | <0.01 | -0.135 | 0.191 | ns |
| **Dist. path** | -0.276 | 0.222 | ns | -1.603 | 0.194 | <0.001 | 0.235 | 0.282 | ns | -1.651 | 0.297 | <0.001 |
| **Dist. fence** | 0.316 | 0.089 | <0.001 | 0.247 | 0.083 | <0.01 | 0.306 | 0.127 | <0.05 | -0.027 | 0.144 | ns |
| **Intercept (red.)** | -4.718 | 2.019 | <0.05 | -2.421 | 0.872 | <0.01 | -5.274 | 1.820 | <0.01 | -4.508 | 0.834 | <0.001 |
| **Dist. shelter** | 0.100 | 0.148 | ns | 0.460 | 0.155 | <0.01 | 0.523 | 0.221 | <0.05 | 0.467 | 0.251 | ns |

**Supplementary Table 5: Model estimates, standard errors, and p values for the GLMMs for buffalos.** The first column contains the covariates. Each model is represented in three columns (e.g. model of feeding buffalos in the summer is in columns 2,3 & 4). Columns with “EST” show model estimates, columns with “SE” show standard errors and columns with “p” show the p-value of the variable in the given model. P-values are non-significant when p >0.05, indicated by “ns”.

| **Variable** | **Feeding** | | | | | | **Non-feeding** | | | | | |
| --- | --- | --- | --- | --- | --- | --- | --- | --- | --- | --- | --- | --- |
|  | **Summer** | | | **Winter** | | | **Summer** | | | **Winter** | | |
|  | **EST** | **SE** | **p** | **EST** | **SE** | **p** | **EST** | **SE** | **p** | **EST** | **SE** | **p** |
| **Intercept (full)** | -3.813 | 0.843 | <0.001 | -2.474 | 0.811 | <0.01 | -4.416 | 0.872 | <0.001 | -3.996 | 0.944 | <0.001 |
| **EVI** | -0.096 | 0.076 | ns | 0.777 | 0.089 | <0.001 | 0.068 | 0.110 | ns | 0.542 | 0.177 | <0.01 |
| **TWI** | -0.026 | 0.075 | ns | 0.230 | 0.072 | <0.01 | 0.100 | 0.107 | ns | -0.303 | 0.167 | ns |
| **Dist. pond (a.)** | -1.600 | 0.276 | <0.001 | 0.281 | 0.227 | ns | -1.025 | 0.363 | <0.01 | 1.416 | 0.425 | <0.001 |
| **Dist. ponds (n.)** | -0.318 | 0.112 | <0.01 | -0.554 | 0.114 | <0.001 | -0.607 | 0.172 | <0.001 | -0.884 | 0.227 | <0.001 |
| **Dist. path** | 0.340 | 0.227 | ns | -0.615 | 0.217 | <0.01 | 0.024 | 0.311 | ns | -0.761 | 0.457 | ns |
| **Dist. fence** | 0.690 | 0.082 | <0.001 | 0.761 | 0.079 | <0.001 | 1.300 | 0.125 | <0.001 | 1.646 | 0.171 | <0.001 |
| **Intercept (red.)** | -3.290 | 0.822 | <0.001 | -2.225 | 0.807 | <0.01 | -4.399 | 0.908 | <0.001 | -3.075 | 0.921 | <0.001 |
| **Dist. shelter** | 0.211 | 0.166 | ns | -0.567 | 0.153 | <0.001 | 0.228 | 0.245 | ns | -0.812 | 0.285 | <0.01 |
